# Manganese Oxide Biomineralization Provides Protection against Nitrite Toxicity in a Cell-Density-Dependent Manner

**DOI:** 10.1128/AEM.02129-18

**Published:** 2019-01-09

**Authors:** Christian Zerfaß, Joseph A. Christie-Oleza, Orkun S. Soyer

**Affiliations:** a School of Life Sciences, University of Warwick, Coventry, United Kingdom; b Warwick Integrative Synthetic Biology Centre (WISB), University of Warwick, Coventry, United Kingdom; University of Toronto

**Keywords:** *Roseobacter*, biomineralization, metal recovery, microbial ecology, reactive oxygen species, respiration

## Abstract

Manganese biomineralization is a widespread process among bacteria and fungi. To date, there is no conclusive experimental evidence for how and if this process impacts microbial fitness in the environment. Here, we show how a model organism for manganese oxidation is growth inhibited by nitrite, and that this inhibition is mitigated in the presence of manganese. We show that such manganese-mediated mitigation of nitrite inhibition is dependent on the culture inoculum size, and that manganese oxide (MnO_X_) forms granular precipitates in the culture, rather than sheaths around individual cells. We provide evidence that MnO_X_ protection involves both its ability to catalyze nitrite oxidation into (nontoxic) nitrate under physiological conditions and its potential role in influencing processes involving reactive oxygen species (ROS). Taken together, these results demonstrate improved microbial fitness through MnO_X_ deposition in an ecological setting, i.e., mitigation of nitrite toxicity, and point to a key role of MnO_X_ in handling stresses arising from ROS.

**IMPORTANCE** We present here a direct fitness benefit (i.e., growth advantage) for manganese oxide biomineralization activity in Roseobacter sp. strain AzwK-3b, a model organism used to study this process. We find that strain AzwK-3b in a laboratory culture experiment is growth inhibited by nitrite in manganese-free cultures, while the inhibition is considerably relieved by manganese supplementation and manganese oxide (MnO_X_) formation. We show that biogenic MnO_X_ interacts directly with nitrite and possibly with reactive oxygen species and find that its beneficial effects are established through formation of dispersed MnO_X_ granules in a manner dependent on the population size. These experiments raise the possibility that manganese biomineralization could confer protection against nitrite toxicity to a population of cells. They open up new avenues of interrogating this process in other species and provide possible routes to their biotechnological applications, including in metal recovery, biomaterials production, and synthetic community engineering.

## Abstract (precis)

We present here a direct fitness benefit (i.e., growth advantage) for manganese oxide biomineralization activity in Roseobacter sp. strain AzwK-3b, a model organism used to study this process. We find that strain AzwK-3b in a laboratory culture experiment is growth inhibited by nitrite in manganese-free cultures, while the inhibition is considerably relieved by manganese supplementation and manganese oxide (MnO_X_) formation. We show that biogenic MnO_X_ interacts directly with nitrite and possibly with reactive oxygen species and find that its beneficial effects are established through formation of dispersed MnO_X_ granules in a manner dependent on the population size. These experiments raise the possibility that manganese biomineralization could confer protection against nitrite toxicity to a population of cells. They open up new avenues of interrogating this process in other species and provide possible routes to their biotechnological applications, including in metal recovery, biomaterials production, and synthetic community engineering.

## INTRODUCTION

A large variety of biominerals based on different cations (e.g., iron, manganese, calcium) and anions (e.g., carbonates, oxides, phosphates) are deposited by different microorganisms (1). One of these is manganese oxide (2–5), which is deposited by the oxidation of soluble Mn^II^. Microbial Mn^II^ oxidation received attention with the discovery of polymetallic, manganese-rich, biogenic deep-sea nodules, which have been shown to harbor both manganese-oxidizing and manganese-reducing organisms (6). While it is suggested that such nodules could potentially be mined for rare earth elements, and the associated metal-active organisms used in biotechnology of metal recovery (2, 3, 5–8), it remains unclear in many cases why organisms carry out such metal oxidizing and reducing activities. In the case of metal-reducing organisms, it has been shown that metabolic energy can be gained under anaerobic conditions from using metal oxides (i.e., manganese, iron, or others) as alternative terminal electron acceptors (9–11). Some metals can be oxidized by microbes and act as an inorganic energy source for so-called chemolithotrophic growth, as in the case of iron lithotrophy (12). While it has been suggested that manganese oxidation can also be used as a chemolithotrophic source of energy (2), little experimental evidence has been found. In most cases studied, Mn oxidation is not used as a lithotrophic source of energy and, hence, evolutionary advantages of this process are not well understood (2, 7, 8). Two running hypotheses for nonlithotrophic manganese oxidation are that the resulting manganese oxides (MnO_X_) can (i) increase accessibility of organic nutrients or (ii) protect microbes from potentially toxic compounds and superoxide stress (13, 14). The validity of the former hypothesis remains to be tested conclusively. MnO_X_ has been shown to react with complex organic (i.e., humic) substances (15), but it is not clear if the resulting organic products from such reactions are utilized by microbes. It has been suggested that certain fungi employ ligand-stabilized Mn^III^ to oxidize recalcitrant litter (16), but these studies were not performed with single (defined) carbon/energy sources. The latter hypothesis regarding the protective potential of MnO_X_ remains unproven to date for metal toxicity (2, 7). It was shown that MnO_X_ can mediate a protection against superoxides in Pseudomonas species (14), but it is not clear how significant this benefit is, given that these and other Mn-oxidizing species also possess specific superoxide-scavenging enzymes, such as catalases and superoxide dismutases (17–19). It has been suggested that MnO_X_ precipitates can act as strong sorbents of heavy metals, hence mitigating the toxic effects of such metals on microorganisms, but this has yet to be tested in a biological context (2). Taken together, the biological significance of microbial manganese oxidation remains largely a paradox, as no clear benefits have been demonstrated.

**FIG 1:**
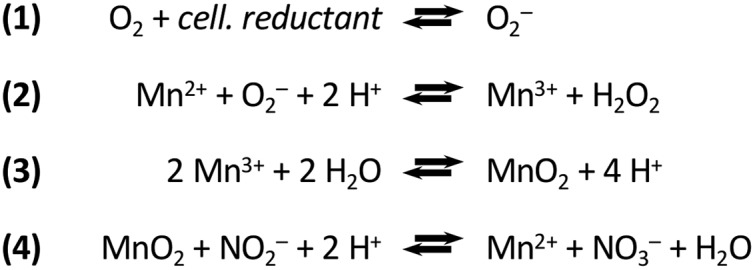
Biological oxidation of manganese via superoxide and nitrite oxidation by the product manganese oxide. These reactions are taken from references 24 (manganese oxidation) and 47 (nitrite oxidation). Note that only representative reactions are presented. For instance, the text refers to a mixed oxide (MnO_X_), while this reaction scheme simplifies to MnO_2_. The cellular reductant (cell. reductant) which serves as electron donor for superoxide production is not unambiguously identified.

In recent years, Roseobacter sp. strain AzwK-3b emerged as a model organism to study the generation of MnO_X_ (20). AzwK-3b is a bacterium that shows significant manganese-oxidizing activity *in vitro* when grown in a complex (rich) K medium (20) and defined (acetate-fed) J medium (21). This activity was shown to be mediated by a secreted exoenzyme—a heme-type oxidase—that can catalyze the *in vitro* generation of superoxides from NADH and oxygen (22) (this and later reactions are shown in Fig. 1), demonstrating the use of biological reductive energy equivalents. The resulting superoxide can in turn facilitate the Mn^II^ oxidation into Mn^III^, which undergoes further disproportionation to result in MnO_2_ (22–26) or, more specifically, mixed-valence-state MnO_X_. While NADH was a suitable electron donor for the *in vitro* superoxide production by heme peroxidase, the natural reducing agent and the way it is delivered are not known. It has been suggested that the heme peroxidase might be loosely membrane bound (27), which would mean that electrons could be shuttled from cytoplasmic reductive metabolites to the heme peroxidase, e.g., via membrane proteins, although this would imply that the natural site of superoxide production (and subsequent manganese oxidation) would be in the immediate proximity of the cell. Since heme peroxidases are also found in culture supernatants (22), an extracellular reaction would require that electron donor metabolites are also secreted, which would imply a considerable investment for AzwK-3b. Thus, these mechanistic findings strongly suggest that AzwK-3b is making a significant metabolic investment into production of MnO_X_ in the form of secreted enzymes and possibly also reductive energy-donating metabolites. Furthermore, strain AzwK-3b’s cellular and excreted proteome is shown to be different when grown in the presence or absence of Mn, while it is notable that the heme peroxidase described above was not found to be differentially expressed (28). It is currently not clear how and if the metabolically costly process of extracellular Mn oxidation benefits individual cells and how it could have been maintained over evolutionary time scales.

In an attempt to better understand any ecologically relevant “fitness” impacts of manganese oxidation, we have studied the physiology of Roseobacter sp. AzwK-3b in more detail. While we did not find any significant difference in growth rate between manganese-free and manganese-supplemented media, we found that the manganese-oxidizing activity of Roseobacter sp. AzwK-3b supports growth of the bacterium at nitrite concentrations that fully prevent growth in a manganese-free culture. MnO_X_ formed as granules dispersed among cells, and its nitrite-inhibition mitigation effects showed a significant population size effect, suggesting a “community commodity” nature of this compound. Mechanistically, we show that biogenic MnO_X_ was able to catalyze nitrite oxidation into nitrate under physiological conditions (according to reaction 4 in Fig. 1) and that the mitigation of nitrite inhibition was also affected by NADH. These results suggest that the ability of MnO_X_ to alleviate nitrite toxicity relates to providing catalytic scavenging of reactive oxygen species (ROS) within the environment, whose effect can be leveraged by nitrite.

## RESULTS

To study the role of manganese oxidation in microbial fitness we have focused here on Roseobacter sp. AzwK-3b, which has recently emerged as a model organism for this process (2, 8). We refer to the oxidation product as MnO_X_, since biogenic manganese oxides are usually precipitates with mixed manganese oxidation states, particularly Mn^II^, Mn^III^, and Mn^IV^ (2, 29). AzwK-3b has been shown to oxidize manganese to MnO_X_ by means of an exoenzyme and reductive energy (e.g., NADH *in vitro*) and potentially involving an elaborate redox reaction path (22–26). We first attempted to identify fully defined growth conditions for this bacterium, which has been studied to date in complex and lean K media (20), both of which contained undefined complex ingredients, such as peptone or vitamin mixtures and yeast extract (20, 30) or standard vitamin supplements (21). Through systematic analysis of medium composition, we have created a minimal defined medium that supports AzwK-3b growth (from now on referred to as modified artificial seawater medium, ASW_m_) (Table 1) and that has revealed the requirement for five specific vitamin supplements for growth (see Fig. S1 in the supplemental material). Given this defined culture medium, we were then able to interrogate the impact of manganese on the growth of AzwK-3b.

**TABLE 1:** Detailed composition of the defined AzwK-3b growth medium, ASW_m_^*a*^

Compound	Concn
Base salts (1× AzwK-3b medium)	
Sodium chloride (NaCl) (mM)	200
Ammonium chloride (NH_4_Cl) (mM)	8.82
Potassium chloride (KCl) (mM)	6.71
Di-potassium hydrogen phosphate (KH_2_PO_4_) (μM)	131
Magnesium sulfate (MgSO_4_) (mM)	14.2
Magnesium chloride (MgCl_2_) (mM)	9.84
Calcium chloride (CaCl_2_) (mM)	3
Tris (mM)	1.1
pH of the medium	8
Trace metal solution (1,000×)	
Copper chloride (CuCl_2_) (μM)	32
Zinc sulfate (ZnSO_4_) (μM)	765
Cobalt chloride (CoCl_2_) (μM)	169
Sodium molybdate (Na_2_MoO_4_) (mM)	1.65
Boric acid (H_3_BO_3_) (mM)	46.3
Nickel chloride (NiCl_2_) (mM)	4.2
Sodium tungstate (Na_2_WoO_4_) (μM)	243
Sodium selenite (Na_2_SeO_3_) (μM)	228
Additional (1,000×) supplement solutions	
Iron chloride (FeCl_3_; prepared in 10 mM HCl, balanced with extra 10 mM NaOH solution) (mM)	10.4
EDTA (pH 8.0; sodium salt) (mM)	1.34
Manganese chloride (MnCl_2_, only added where desired) (mM)	200
Vitamin supplement (1,000×)	
Biotin (μM)	82
Pyridoxine hydrochloride (μM)	484
Thiamine hydrochloride (μM)	148
Riboflavin (μM)	133
Nicotinic acid (μM)	406

a The medium was developed starting from artificial seawater (ASW) (35), with extra trace metals taken from references 9 and 94 and a 5-vitamin solution identified starting from Wolfe’s vitamin mixture (95).

### Manganese oxidation has negligible impact on growth rate.

**FIG 2:**
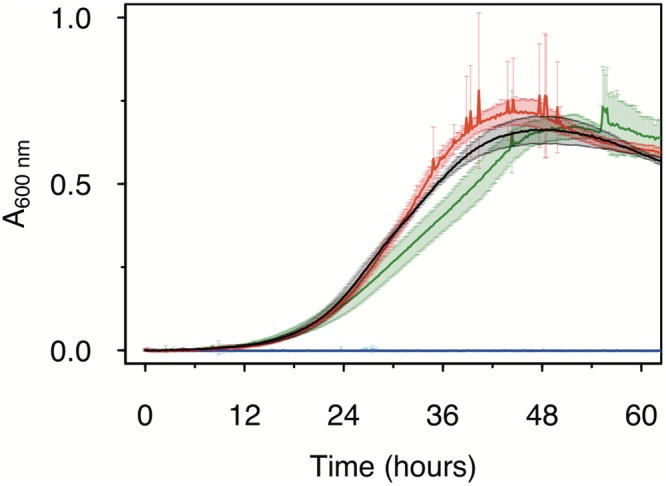
Effect of Mn^II^ on the growth of Roseobacter sp. AzwK-3b in the defined growth medium (Table 1). The concentrations of manganese were 0 μM (black), 200 μM (red), and 500 μM (dark green), with no growth (zero line) in the respective noninoculated controls (blue, magenta, and light blue). Cultures were grown in a 96-well plate (200-μl culture), with shaking, and absorbance measurements were taken every 10 min (see Materials and Methods).

Despite potentially significant costs associated with exoenzyme secretion and the investment of reductive energy equivalents for superoxide generation, we did not find any substantial difference in growth rates and steady-state population sizes with increasing Mn^II^ concentration for cultures grown with 25 mM acetate (Fig. 2). A slightly lower growth rate at the highest manganese concentration (500 μM) was observed, but it was difficult to ascertain this effect, as both MnO_X_ particles and cells coaggregating with those particles could have interfered with the absorbance measurements. The slightly reduced growth rate at 200 μM Mn^II^Cl_2_ is in line with results of an earlier report on AzwK-3b, where 100 μM Mn^II^ was found to decrease the growth rate in (complex) K medium (20). Other manganese-oxidizing bacteria, such as Erythrobacter sp. strain SD-21 (31, 32) and a marine Bacillus strain (33), were reported to grow better when cultured with a Mn^II^ supplement. In light of these different findings and possible difficulties with growth rate measurements in the presence of manganese precipitation, we cannot be fully conclusive about the growth effects associated with manganese oxidation based on the presented results; however, they are suggestive of low or no impact on growth rate.

### Manganese oxidation mitigates nitrite growth inhibition.

**FIG 3:**
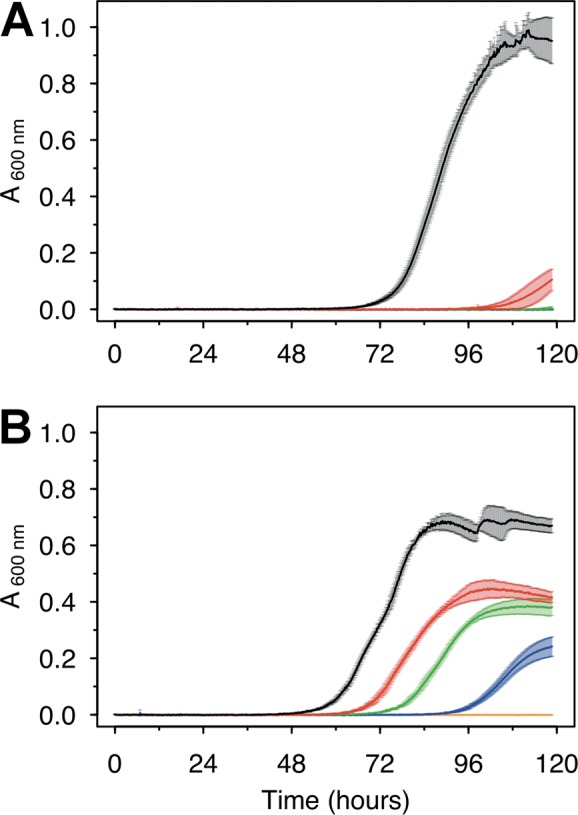
Growth of Roseobacter sp. AzwK-3b cultures in the defined growth medium supplemented with sodium nitrite. Media were prepared without (A) or with (B) 200 μM manganese chloride (Mn^II^Cl_2_). Nitrite concentrations were 0 mM (black), 0.25 mM (red), 0.5 mM (green), 1 mM (dark blue), and 2.5 mM (light blue). All conditions were tested in triplicate, and the growth curves represent averages and their standard deviations (see Materials and Methods).

With growth effects being limited, a possible alternative explanation for a positive role of manganese oxidation is a protective effect against inhibitors or stresses (2, 13). Here, we evaluated this hypothesis for nitrite. Nitrite is commonly found in the environment, where it results from the reduction of nitrate, a key terminal electron acceptor utilized by many microbes (34). We found that nitrite inhibited the growth of AzwK-3b in manganese-free cultures, where already as low as 0.25 mM nitrite prevented growth of AzwK-3b (Fig. 3A). To rule out a salinity effect, different concentrations of sodium chloride were tested (200 mM [default in ASWm] to 428 mM NaCl [default in original ASW medium (35)]), and AzwK-3b grew in all tested conditions (Fig. S2).

With the addition of 200 μM Mn^II^, we found that AzwK-3b is able to grow in the presence of up to 1 mM nitrite (Fig. 3B). Increasing the nitrite concentration still affected both the growth rate and maximal culture density (based on *A*_600_), but this effect was much lower than that in the manganese-free cultures (Fig. 3). To overcome any potential confounding effects of MnO_X_ precipitation on spectroscopic culture density measurements, we additionally quantified acetate consumption as a proxy for growth, using ion chromatography. As expected, manganese-free cultures with 0.25 mM (or higher) nitrite concentrations showed only insignificant decreases in acetate, while the Mn^II^-supplemented cultures showed acetate consumption in accordance with the *A*_600_ measurements (see Fig. S3). These findings confirm that Mn^II^ supplementation allows AzwK-3b to withstand nitrite inhibition.

### Nitrite inhibition relief is a community function that depends on culture size and that is mediated by dispersed, granular MnO_X_ precipitates.

It has been shown that MnO_X_ precipitation by AzwK-3b is mediated by secreted exoenzymes (22). It is not known, however, whether the process of MnO_X_ precipitation occurs primarily on individual cell surfaces, or whether it is a population-level process, with the secreted enzymes conferring the notion of a community commodity (36–39). We hypothesized that these two different scenarios could be distinguished by analyzing population size effects on MnO_X_-mediated mitigation of nitrite inhibition. In particular, we designed an experiment in which cultures pregrown without Mn^II^ are subsequently subcultured into medium with Mn^II^ and nitrite, using different inoculum sizes. We argue that, in the case of MnO_X_-based protection being a process confined to individual cells, there should be no effect of inoculation size.

**FIG 4:**
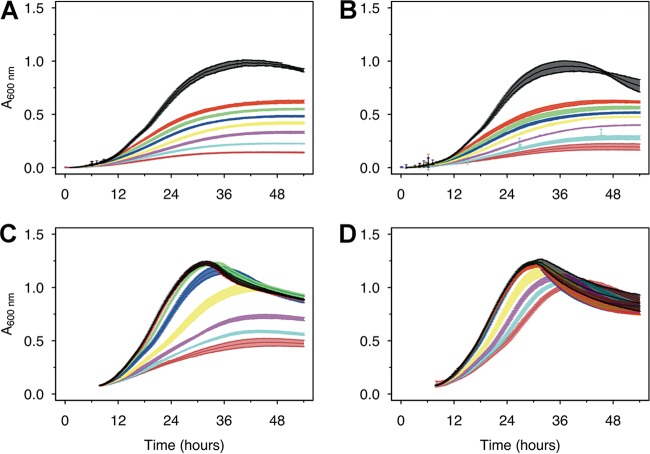
Larger AzwK-3b inocula are less inhibited by nitrite. A preculture without manganese or nitrite was grown and sampled in the exponential growth phase (Fig. S4) to prepare inocula from a very early time point in the exponential phase (IT1, panels A and B) and from a later time point (IT2, panels C and D; both sampled in the first third of the exponential phase). These inocula were diluted 1:1 with fresh medium and tested for growth at different nitrite concentrations (see below for color code) without (A, C) or with (B, D) 200 μM Mn^II^Cl_2_ supplement. The nitrite concentrations were as follows: black, control/no nitrite; red, 0.25 mM nitrite; green, 0.5 mM nitrite; blue, 1 mM nitrite; yellow, 2 mM nitrite; magenta, 5 mM nitrite; light blue, 7.5 mM nitrite; and dark red, 10 mM nitrite. Growth curves show the averages and standard deviations over a triplicate analysis (see Materials and Methods).

We found that MnO_X_-based protection against nitrite inhibition was dependent on inoculum size (Fig. 4). A preculture was grown without nitrite and manganese and, from this, inocula were generated at two different time points within the first third of the exponential phase (labeled IT1 and IT2 in Fig. S4). When these inocula were subjected to nitrite in the main culture, the earlier, low-density inoculum, IT1, was inhibited by nitrite regardless of the presence or absence of Mn^II^ (Fig. 4A and B), while manganese-mediated mitigation of nitrite inhibition was clearly evident for the larger, high-density inoculum, IT2 (Fig. 4C and D). In the IT1 cultures, half of the acetate was unused at 0.25 mM nitrite, and, gradually, more acetate resided with increasing nitrite concentration (Fig. S5). In the IT2 cultures with Mn^II^ supplementation, however, acetate was completely removed at all nitrite levels below 2.5 mM, and only 25% to 50% of acetate remained at 5 to 10 mM nitrite. In the control samples (no inoculation) there was no change in acetate concentration, ruling out any cross-activity with manganese.

**FIG 5:**
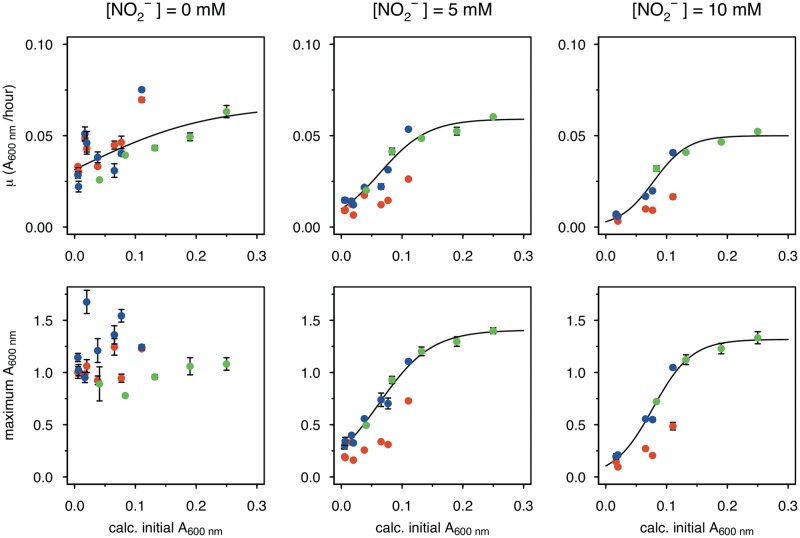
Inoculum size effect on MnO_X_-mediated mitigation of nitrite inhibition. Data from different AzwK-3b growth experiments of similar type (large inocula; see Materials and Methods) were analyzed for the maximum *A*_600_ (bottom row) and growth rate (top row) by fitting the growth curves. Each condition was done in three technical replicates (note that error bars are not visible in some cases due to only small differences). Nitrite concentrations of the main cultures are indicated as headings of the figure columns. The *x* axes show the calculated *A*_600_ values of the initial cultures after diluting them 1:1 from the precultures, while the *y* axes show the maximum *A*_600_ and maximum growth rate values as calculated with the Gompertz model (91, 92) (see Materials and Methods). The colors represent different conditions, as follows: red, neither preculture nor main culture contained manganese; blue, preculture without and main culture with manganese; and green, both preculture and main culture with manganese. The black curve is a sigmoidal fit (logistic model) from the R package Grofit (91), for the results of the combined blue and green data set, where the nitrite-exposed main cultures all contained manganese.

Rather than a true population size effect, these observed inoculum effects could be due to cells from the Mn-free, early-phase precultures not having “turned on” expression of exoenzymes required for MnO_X_ precipitation. To rule out this possibility, we performed an additional experiment, where the precultures were already grown with 200 μM Mn^II^. Using this preadapted culture, inocula were again prepared by sampling at different growth time points (IT1 to IT4 in Fig. S6A). Cultures grown from these different inocula displayed much weaker inhibition by increasing nitrite concentrations up to 10 mM (Fig. S6B) and were able to consume acetate (Fig. S6C), yet there were still inoculum size effects on overcoming nitrite inhibition (Fig. 5, green). Interestingly, the extent of this effect seems similar to that observed with inocula originating from precultures grown without Mn^II^ but supplied with Mn^II^ after subculturing into nitrite-containing medium (Fig. 5, blue). In particular, at 5 and 10 mM nitrite, maximum growth rate (and final density) data from these two treatments can all be fitted on to a single (sigmoidal) curve that describes the relation between these data and initial inoculum density (Fig. 5, black line). This shows that the presence of Mn^II^ in the preculture does not impact the dynamics of the process but rather allows the main-culture populations to grow to a higher density under a given nitrite level. In the absence of Mn^II^ in both precultures and main cultures, a much denser inoculum was required to achieve growth at a given nitrite level, and even then, both growth rate and final growth density were lower than those measured in the presence of Mn^II^. Under this condition, there was still a density dependence of the nitrite effect.

**FIG 6:**
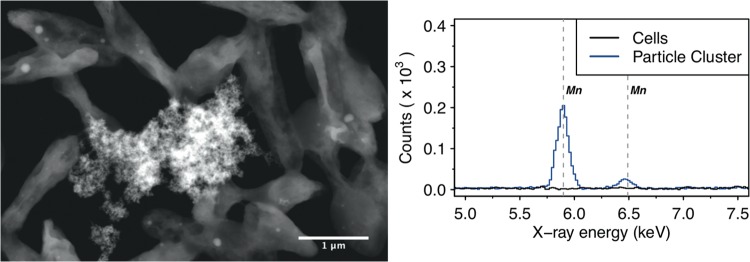
Scanning transmission electron micrograph (left figure, high-angle annular dark field) of (granular) manganese-containing precipitate (center) surrounded by AzwK-3b cells, and associated energy-dispersive X-ray spectroscopic analysis (right figure) in this location. Only the energy range containing the manganese-specific X-ray energies at 5.90 keV (K_α_^I^) and 6.49 keV (K_β_^I^) is shown, and the manganese transitions are indicated by vertical gray dashed lines.

These results suggest that MnO_X_ precipitation is a community-level function. To further elaborate on this result, we explored the microstructure of the AzwK-3b cultures in the presence of Mn^II^. Analysis of cultures using electron microscopy revealed MnO_X_ precipitates as granules dispersed within the culture and attaching to clusters of cells, rather than forming sheaths around individual cells (as seen in some other cases of metal oxide precipitations [40]) (Fig. 6, left). Employing electron-dispersive X-ray spectroscopy, we confirmed that these granular structures contained manganese, while no manganese was detected in locations with cells only (i.e., without granular structures; Fig. 6, right).

### MnO_X_-mediated nitrite protection involves redox reactions and oxygen radicals.

After establishing the community-level functionality of biogenic MnO_X_ as a protective agent against nitrite, we next wanted to evaluate the mechanistic basis of this function in the context of nitrite toxicity. While multiple mechanisms of nitrite toxicity have been reported (41, 42), two key reactive species are usually implicated, i.e., free nitrous acid (43) and peroxynitrite. The former forms through protonation of nitrite, while the latter forms from the reaction of nitrite with hydrogen peroxide (44–46). Thus, two nonexclusive possible mechanisms of MnO_X_ relief on nitrite toxicity are (i) that MnO_X_ catalyzes oxidation of nitrite to nitrate (a reaction that has been shown to be chemically feasible under low-pH conditions [47]) and thereby avoids formation of either free nitrous acid or peroxynitrite, or (ii) that MnO_X_ catalyzes degradation of hydrogen peroxide and thereby avoids the reaction of this compound with nitrite to form peroxynitrite.

**FIG 7:**
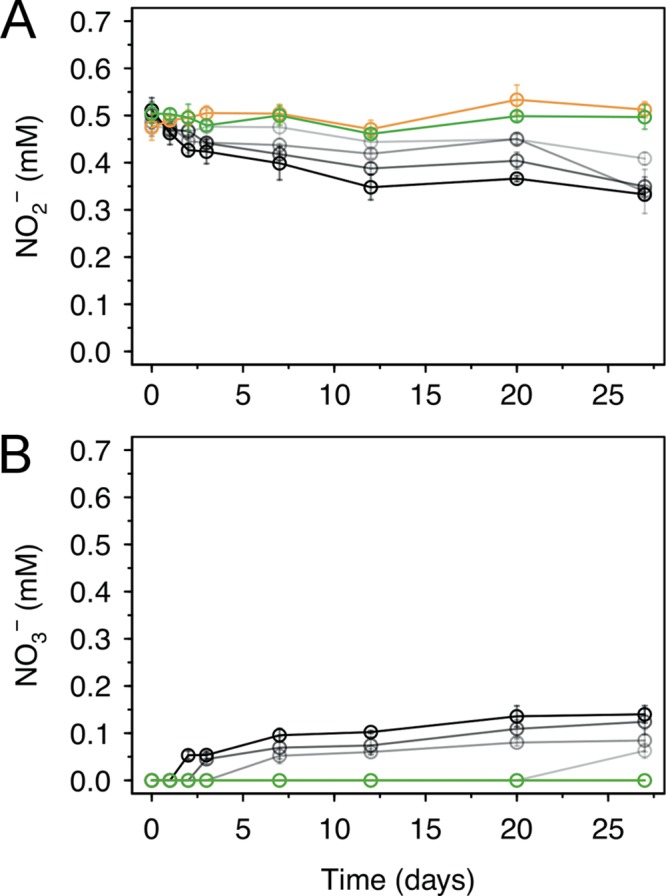
Oxidation of nitrite by biogenic manganese oxide (MnO_X_) produced in cell-free culture supernatant of AzwK-3b. The figure shows the concentrations over time of nitrite (A) and nitrate (B), as determined by ion chromatography (note that concentrations were corrected for the IC peak from chloride, to account for evaporation during the experiment). As controls, samples without MnO_X_ (green) or with MnO_2_ powder (orange) were included in the experiment (see Materials and Methods). The samples with AzwK-3b cell-free manganese oxide contained (from gray to black) 0.2, 0.5, 1, and 2 mM manganese oxide equivalent (see Materials and Methods).

To see if AzwK-3b-generated MnO_X_ can catalyze nitrite oxidation under physiological conditions, we collected it from culture supernatants and evaluated its reactivity with nitrite in our ASW_m_ (pH = 8.0). Over 27 days, we found that nitrite oxidation by biogenic MnO_X_ occurred in a dose-dependent manner, while neither synthetic MnO_2_ powder nor the MnO_X_-free solution showed any significant nitrite oxidation (Fig. 7A). The trend of nitrite oxidation matched with that of nitrate production (Fig. 7B), thus confirming the assumed reaction pathway of nitrite oxidation into nitrate (47). Taking into account the difficulties of accurately determining the amount of precipitated MnO_X_ that was added into the nitrite assay, we can still estimate that the highest MnO_X_ levels were at least 1 to 2 mM (with respect to Mn). This presents a stoichiometric minimum 2-fold excess over nitrite (at 0.5 mM) and hence enough for complete nitrite oxidation. The fact that this reaction did not proceed further than an oxidation of ∼0.18 mM nitrite (i.e., ∼35%) indicates either that the biogenic MnO_X_ was only partially reactive or that its reactivity reduced over time (as is known to be the case for synthetic manganese oxides [2, 13]). Sample pH remained relatively stable with the biogenic MnO_X_, while samples without manganese and with synthetic MnO_2_ reached pHs of 6.9 and 6.8, respectively, at the end of the experiment (from an initial pH of the medium of 8.2). This acidification of the control samples might be due to carbon dioxide dissolution; carbon dioxide might have been buffered in the samples with biogenic MnO_X_ due to proton consumption during nitrite oxidation or due to coprecipitated organic solutes (polymers, proteins) from the cell-free supernatant.

These findings confirmed that biogenic MnO_X_ was capable of oxidizing nitrite under physiological conditions and prompted us to test MnO_X_-mediated nitrite oxidation directly in AzwK-3b cultures. We found some evidence for decreasing nitrite concentration in different cultures tested, but this was not significant (Fig. S7), and some decrease was also seen in the manganese-free cultures (indicating possible measurement effects in the solution). If nitrite oxidation was the main mechanism of MnO_X_-mediated protection *in vivo*, these cultures would have been expected to oxidize most of the nitrite present in the medium. Thus, we conclude that under our experimental conditions, nitrite oxidation was only a potential contributing factor.

**FIG 8:**
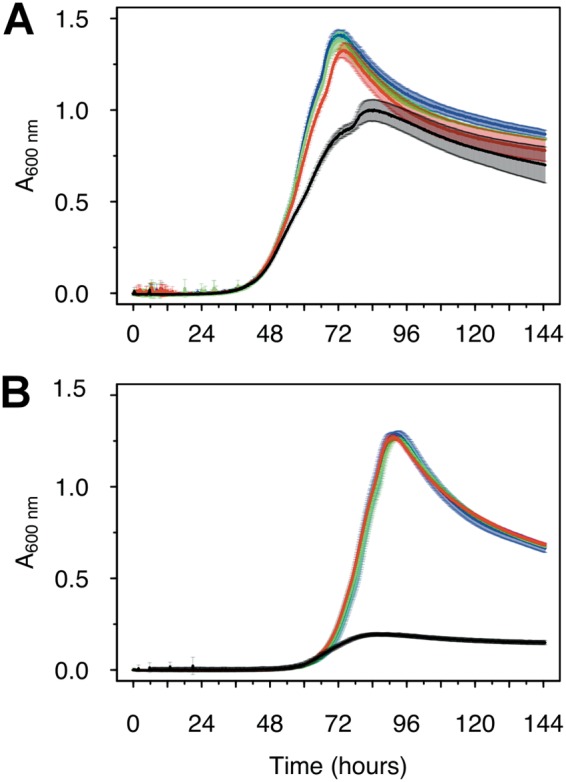
Reductive power (NADH) mitigates the growth inhibitory effects of nitrite in AzwK-3b. Cultures (preculture and main culture without manganese) were grown in the absence (A) and presence (B) of 5 mM nitrite and supplement of 0, 50, 100, and 200 μM NADH (black, red, green, and blue) at the start of the culture.

A plausible alternative mechanism of MnO_X_-mediated nitrite inhibition relief could be related to formation of reactive peroxynitrite, which has been shown to be highly toxic to bacteria (45, 46, 48, 49) and which can form (particularly) at low pH from the reaction of hydrogen peroxide with nitrite (44). If peroxynitrite is the main species underpinning nitrite toxicity, then MnO_X_ protection against nitrite could be due to its ability to degrade hydrogen peroxide, thereby reducing the rate of peroxynitrite formation. The reactivity of MnO_X_ toward hydrogen peroxide has been chemically demonstrated (44, 50–57), but never shown or tested in a biological context. Here, we hypothesized that if these types of redox reactions were involved in MnO_X_-mediated mitigation of nitrite inhibition, the process dynamics can be modulated with the introduction of additional hydrogen peroxide or NADH (which can help increase the rate of MnO_X_ formation [23], but which can also be directly involved in hydrogen peroxide reduction through peroxidase-catalyzed reactions [17–19, 58, 59]). To test this hypothesis, we again grew precultures of AzwK-3b without Mn^II^ and subcultured these in medium containing Mn^II^ and nitrite, but at the same time also spiking in hydrogen peroxide or NADH. Hydrogen peroxide spiking did not show any effect on nitrite inhibition or its release by Mn^II^ supplementation (Fig. S8), possibly due to spiked hydrogen peroxide being cleared primarily through additional peroxidases rather than impacting MnO_X_-mediated process dynamics. In line with this hypothesis, spiking NADH resulted in full mitigation of the nitrite-inhibitory effect (even without Mn^II^) (Fig. 8). This suggests that nitrite toxicity relates to peroxynitrite formation via hydrogen peroxide, which can be decomposed by MnO_X_ (as shown before [44, 50–57]) or NADH-utilizing peroxidases (which are shown to be present in Roseobacter species, including strain AzwK-3b [22, 27]; see also Table S1).

## DISCUSSION

Manganese biomineralization into MnO_X_ is widespread among bacteria, but there is no clarity about its possible functional roles. Here, we developed a defined growth medium for the manganese-oxidizing model organism Roseobacter sp. AzwK-3b and demonstrated that, in a laboratory setting, this organism’s strong growth inhibition by nitrite is mitigated through its ability to precipitate biogenic MnO_X_. While our experiments were undertaken in an artificial lab environment, these findings provide direct evidence for the impact of MnO_X_ on an organism’s growth, thus raising the possibility of a positive fitness effect and a possible ecological/evolutionary explanation for the costly process of MnO_X_ oxidation.

Interestingly, we also show that the MnO_X_-mediated mitigation of nitrite toxicity is dependent on population size, and that MnO_X_ forms dispersed granules that are attached to clusters of cells in the population. These observations, combined with the established role of exoenzymes in the formation of MnO_X_ precipitates, suggest that these granules provide a community function to AzwK-3b and allow cultures grown to sufficient density in the presence of manganese to become resistant to the inhibitory effects of nitrite. Our attempts to elucidate the mechanistic basis of this functionality showed that biogenic MnO_X_ can oxidize nitrite to nitrate (under conditions in which synthetic MnO_2_ cannot). Together with the known ability of MnO_X_ to degrade hydrogen peroxide (44, 50–57), these findings show that biogenic MnO_X_ can inhibit the two key routes to the formation of reactive nitrite species.

While mitigation of nitrite inhibition might not be the only evolutionary advantage of MnO_X_ oxidation in AzwK-3b or other manganese-oxidizing species, it is definitely an ecologically relevant function. Nitrite is a known inhibitor in the environment (41, 42, 60), including in wastewater treatment applications (43). In soil, reported nitrite concentrations are in the range of low μmol/kg or μM (61, 62), although they can peak to higher than 0.5 mmol/kg with agricultural nitrogen fertilization (61). In biofilms, where diffusion is inhibited, oxygen is shown to rapidly diminish (63–65), which can favor anaerobic metabolism, including nitrate respiration to nitrite (66). Furthermore, biofilms are shown to preferentially select for and accumulate ions such as phosphate and nitrite (67–69). For example, in freshwater lake biofilms, the annual variation range for nitrite was found to be in the range of μM to mM (i.e., 1,000-fold) in biofilms (68). In the case of AzwK-3b, these physical and ecological processes can be highly relevant, as this species was isolated from an “agriculturally impacted, shallow salt marsh” (20), where nitrite (among other nitrogen species) can occur due to microbial conversion of nitrogen fertilizers (61, 70–72). It is also interesting to note that oceanic manganese-rich modules are found to contain both manganese-oxidizing and manganese-reducing bacteria (6), with current-day representatives of the latter group, such as Shewanella oneidensis (9), also being nitrate reducers (73–75). Thus, these nodules also can or have harbored high levels of nitrite, creating environments that select for manganese oxidation.

Our study opens up additional investigations into the mechanism of nitrite toxicity and the role of MnO_X_ oxidation in it. Multiple mechanisms of nitrite inhibition have been reported (41, 42), and a key role for free nitrous acid (i.e., protonated nitrite) (43) and peroxynitrite, from nitrite and hydrogen peroxide (44–46), is proposed. Both molecules can prevent chemiosmotic coupling, and they are primarily formed at low pH (nitrite is often found to inhibit bacterial survival at a pH of <7 [45, 46]). Indeed, low pH can arise within the cellular microenvironment; energy metabolism coupled to chemiosmosis generates a proton motive force that can enrich the proton concentration at the charged membrane surface (values as low as pH 5.5 to 6.5 are discussed). This local low-pH environment can be further stabilized and inhibited from equilibration with the bulk due to an electrostatic barrier imposed by water layering (76, 77). Additionally, respiratory activity can increase hydrogen peroxide in the same cellular microenvironment (18, 48, 49, 58, 78–85), which can facilitate nitrite conversion to peroxynitrite. Interestingly, these local conditions could be avoided through the presence of MnO_X_, which can degrade hydrogen peroxide and catalyze the oxidation of nitrite to nitrate, which is a proton-consuming process with an increased rate at low pH (47). The latter mechanism is confirmed here under physiological conditions, as we show that biogenic MnO_X_ can also catalyze nitrite oxidation at pH 8.

MnO_X_-mediated hydrogen peroxide degradation as a mechanism to prevent peroxynitrite formation remains to be fully confirmed. Our experiments with spikes of hydrogen peroxide did not alter the gross dynamics of MnO_X_-mediated nitrite inhibition relief, but this could be due to the design of these experiments, with hydrogen peroxide delivered in single doses rather than being delivered in a controlled manner in the vicinity of the cells. A single dose could have been readily dealt with by additional peroxidases without altering MnO_X_-mediated effects. On the other hand, our observation that the nitrite stress is fully mitigated in NADH-supplemented cultures (even in the absence of MnO_X_) lends support to the idea that nitrite stress is mediated primarily through formation of peroxynitrite. In that case, the reductive power of NADH could be employed by peroxidases, as well as by MnO_X_, to reduce hydrogen peroxide (17, 58, 59), thereby stopping the formation of peroxynitrite and explaining the observed mitigation effect of NADH.

These possible mechanistic scenarios of nitrite toxicity, and the roles of NADH, peroxidases, and MnO_X_ in mitigating it, can shed light on why and if manganese oxidation is a functional, actively evolved trait or not. In particular, it is not clear why cells that already have several peroxidases, such as those of strain AzwK-3b (22, 27) (Table S1), might invest additional energy in the formation of MnO_X_ precipitates. One possibility is that the formation of MnO_X_ is a mere side effect arising from the microbially generated superoxide (which is widespread among bacteria) (27) reacting with the manganese (Mn^II^) and from the exoenzymes of AzwK-3b simply removing the resulting hydrogen peroxide that would otherwise lead to subsequent reduction of the oxidized manganese (24). An alternative possibility is that manganese oxidation is actively selected for due to the exact reaction mechanisms of ROS scavenging. It has been suggested, for example, that different ROS-scavenging enzymes have different substrate affinities and efficiencies (18). In this context, MnO_X_-mediated scavenging could be preferred under certain ROS concentrations and modes of production. In addition, and unlike peroxidases that require stoichiometric equivalents of reductants, such as, e.g., NADH/NADPH for hydrogen peroxide reduction (18, 19), MnO_X_ in its different oxidation states (II, III, and IV) can, once formed, directly catalyze degradation of hydrogen peroxide without NADH involvement (23, 24, 26, 44, 50–57). The fact that some peroxidases, as well as the AzwK-3b enzyme catalyzing MnO_X_ formation, are exoenzymes (22, 86) could be also highly relevant. The expression of such exoenzymes is a “social trait” that can be exploited by cheating cells that do not invest the costs but reap the benefits (36–39). The presented finding that MnO_X_ forms dispersed granules in the (agitated) liquid culture of strain AzwK-3b shows that, in this case, the ultimate functional effects arising from exoenzyme activity are localized. This kind of localization is a known strategy to stabilize a social trait in the face of evolution of cheating, as seen in exoenzymes with localized actions involved in sugar degradation (87) and metal scavenging (88). Thus, the reductive energy investment into the formation of MnO_X_-mediated protection might be a metabolically less costly strategy that is also socially more stable, compared to, for example, exoenzymes that are freely diffusing.

Within a wider context, our findings are relevant to understanding the different forms of metal mineralization observed in different microorganisms and under different ecological contexts. Given the abundance of microorganisms involved in reactions of the nitrogen cycle, there is indeed potential transient accumulation of nitrite in different environments. It is also possible that MnO_X_ (or other minerals) can provide broader protection against ROS chemistry. For example, manganese oxidation is also observed in spore-forming bacteria (89, 90), fungi, and other microorganisms (as reviewed and shown in references 2 and 40), where a role for nitrite stress remains to be elucidated. Our findings will facilitate such further studies of biomineralizing organisms and their different functional motives and social strategies.

## MATERIALS AND METHODS

### Bacterial strain and culture conditions.

Roseobacter sp. AzwK-3b was obtained from Colleen Hansel (Woods Hole Oceanographic Institution, Falmouth, MA), who isolated the strain (20). Cultures were grown in a defined medium, which was established by modifying the predefined artificial seawater (ASW) medium (35). This modified artificial seawater medium is referred to here as ASW_m_, and its composition is shown in Table 1. ASW_m_ contained sodium acetate as the sole carbon source (at concentrations specified per experiment), 200 mM sodium chloride (instead of 428 mM, as in ASW), ammonium as the nitrogen source (instead of nitrate, as in ASW), and five vitamins that were added as a supplement. In manganese-supplemented ASW_m_, manganese chloride (MnCl_2_) was added to a final concentration of 200 μM. Cultures were grown at 30°C in appropriate (100-ml) Erlenmeyer flasks (shaking at 150 strokes per minute) or 96-well polystyrene plates (Corning, Inc.) closed with a lid and Parafilm (shaking at 300 strokes per minute). For flask cultures, a MaxQ 4000 shaking incubator (Thermo Fisher Scientific) was used. Plates were incubated in a CLARIOstar plate reader (BMG Labtech), and absorbance measurements were done at 600 nm (*A*_600_) and with path length correction, so as to present absorbance per 1 cm.

### Electron microscopy and energy-dispersive X-ray spectroscopy analysis of AzwK-3b cultures.

A culture of AzwK-3b cells (40 ml in 100-ml Erlenmeyer flasks) was inoculated in ASW_m_ without manganese and nitrite and containing 50 mM acetate. After 3 days at 150 strokes per minute shaking at 30°C (by which time the culture reached the stationary phase), dilutions (25× to 200×) were made for a second passage of culture in the same medium, supplemented with 200 μM manganese. After a further 2 days of culturing, samples for electron microscopy (EM) were prepared as follows. Cells from 2.5 ml of culture were harvested by centrifugation (5 min at 5,000 × g), and the supernatant was discarded. From here, several washing and dehydration steps were conducted by resuspending the pellet in different solutions and subsequently centrifuging for 5 min at 5,000 × g (supernatant discarded), as follows: (i) first, pellets were twice resuspended in ASW_m_ base salts (Table 1) (no manganese, no acetate, no ammonium, no nitrite, and no trace metals); (ii) afterwards, samples were resuspended in 200 μl of 70% ethanol, incubated for 1 min, and pelleted by centrifugation; (iii) for a washing-dehydration step, pellets were twice resuspended in 200 μl of 100% ethanol and harvested by centrifugation; and (iv) finally, samples were resuspended in 100 μl of 100% ethanol. This suspension was then applied to transmission electron microscopy (TEM) grids (Lacey carbon film-coated copper grids; Agar Scientific) by pipetting in 1-μl portions (allowed to dry in between) until a total of 2 or 5 μl was accumulated (on different grids). After letting them dry on the bench overnight, grids were analyzed by EM.

EM analysis was done on a Gemini scanning electron microscope (SEM) 500 (Zeiss) equipped with an energy-dispersive X-ray spectroscopy (EDS) X-Max detector (Oxford Instruments). Data analysis was done on the associated AZtec software, which contained the spectral information to identify individual elements. Electron micrographs had the best quality in scanning transmission EM (STEM) mode with a high-angle annular dark-field detector (HAADF). For EDS, the sample needed to be moved, and the HAADF detector had to be withdrawn, so the location of analysis after changing the setup was confirmed by additional SEM recording. The HAADF recording presented in Fig. 6 was recorded at 25 kV and a 4.3-mm working distance, with a ×50,000 magnification. The EDS was recorded at 25 kV, and spectra were accumulated for the same amount of time (40 s for the two locations compared in Fig. 6).

### Large inoculum preparation for nitrite assays.

AzwK-3b cultures were grown in Erlenmeyer flasks (usually 40 ml culture volume in a 100-ml Erlenmeyer flask) in ASW_m_ with 25 mM acetate. The culture absorbance (*A*_600_) was recorded regularly on a Spectronic 200 spectrophotometer (Thermo Fisher) with 1-cm path length polystyrene cuvettes, and inocula were sampled at various stages of the growth curve (e.g., see Fig. S4, S6, and S8 in the supplemental material). This culture was used for inoculation into 96-well plates, which were supplemented by 1:1 dilution with fresh medium supplemented with manganese and/or nitrite and other additives, as described for the particular results shown (legends of Fig. 4 and 6 and Fig. S6 and S8). Where noted (see respective figure captions), the fresh medium used for dilution was also supplemented with NADH or hydrogen peroxide at different concentrations. NADH or hydrogen peroxide was the last additive (to prevent reactions, e.g., between hydrogen peroxide and Mn^II^, before inoculation), and the completed fresh medium was used immediately.

### Growth curve fitting and analysis.

Growth curves were analyzed using the R package Grofit (91), applying the Gompertz growth model (91, 92). Plate reader data (measurements every 10 min) were denoised by averaging over 6 measurements (i.e., hourly averages). The maximum *A*_600_ reached was read directly from the data. For curve fitting, all data obtained later than the maximum *A*_600_, i.e., the decaying growth phase, were removed. Then, the data were read backwards in time to find the first reading that was below 5% of the maximum *A*_600_. This data trimming was done to facilitate the fitting of the Gompertz growth model without bias from different lag phases (which were ignored), or different lengths and scales of decaying phases recorded. From the resulting model, the maximum growth rate, μ (in *A*_600_ [absorbance units] per hour), was recorded.

### Preparation of cell-free bio-manganese oxide.

The procedure was adapted from previous publications using the cell-free supernatant of Roseobacter sp. AzwK-3b cultures grown in complex medium (20, 22–24). AzwK-3b was grown in ASW_m_ supplemented with 50 mM sodium acetate for 9 days, using individual 50- or 100-ml cultures in 100- or 200-ml Erlenmeyer flasks, respectively, at 30°C with shaking (150 strokes per minute). In total, 2 liters of culture was prepared, cells were removed by centrifugation (5 min at 10,000 × g), and the supernatants were combined. From this (cell-free) supernatant, individual samples of 100 or 200 ml were prepared and supplemented with 200 μM manganese chloride (MnCl_2_). Manganese oxidation was allowed to proceed for 5 days at 30°C with shaking (150 strokes per minute), after which the manganese oxide was harvested by centrifugation (5 min at 10,000 × g) from each 50- or 100-ml sample. These were combined and washed by suspending in 25 ml acetate-free ASW_m_ and resedimented by centrifugation. The pellet was brown in appearance and had considerable volume, indicating coprecipitation of organic material (e.g., secreted proteins) from the cell culture supernatant. To estimate the amount of manganese precipitated in the assay, the supernatants from centrifugation and the washing steps were combined, and the residual manganese was determined by the 3,3′,5,5′-tetramethylbenzidine (TMB) assay (93) for soluble manganese. Note that this was not a precise quantification, but it was conclusive enough to allow conservative stoichiometric relations to be inferred. In particular, we inferred that ca. 75% of the 200 μM manganese chloride had been removed from the solution, and this value was used for downstream calculations. The MnO_X_ precipitate was suspended in an appropriate volume of the acetate-free medium to produce a “10 mM” suspension of manganese oxide, and this value is used in this study as an indicator for manganese oxide concentration. The pH was 8.2, which is well in line with the pH 8.0 of the ASW_m_, showing that the suspended manganese oxide did not alter the pH.

### Quantification of nitrite, nitrate, and acetate.

Quantification was done by ion chromatography (IC) on a Dionex ICS-5000+ instrument (Thermo Fisher, UK) equipped with a conductivity detector, potassium hydroxide (KOH) eluent generator, appropriate suppressor, and a Dionex IonPac AS11-HC-4μm anion separation column (2 × 250 mm; Thermo Fisher, UK) with appropriate guard column. Culture samples were filtered with a 0.22-μm polyamide spin filter (Costar Spin-X; Corning, NY) and diluted 10-fold with MilliQ water (resistance [*R*], >18.2 MΩ), of which 2.5 μl was injected for IC separation. The IC was run as a continuous gradient, as follows (flow rate, 0.38 ml/min; column temperature, 30°C; conductivity detector cell temperature, 35°C): −7 to 0 min, 1.5 mM KOH (equilibration); 0 to 8 min, 1.5 mM KOH; 8 to 18 min, increase to 15 mM KOH; 18 to 23 min, increase to 24 mM KOH; 23 to 24 min, increase to 60 mM KOH; and 24 to 30 min, stay at 60 mM KOH. Reference samples with known concentrations were run for calibration. During the course of the experiments, evaporation of the samples was noted (indicated by the increase in the peak area of chloride, which is expected to be unaltered by any biologic means and which therefore should have displayed no concentration change). To correct for this evaporation effect, the concentrations of the analytes of interest were corrected by the same ratio as that obtained from the chloride peak area (from the beginning and endpoint samples of a particular time course experiment).

## Supplementary Material

Supplemental file 1
