# Supplementary material for: Manganese Oxide Biomineralization Provides Protection against Nitrite Toxicity in a Cell-Density-Dependent Manner
Source: Appl Environ Microbiol. 2019 Jan 9;85(2):e02129-18. doi: 10.1128/AEM.02129-18 (PMC6328764; doi:10.1128/AEM.02129-18)
Supplement: Supplemental file 1 [file AEM.02129-18-s0001.pdf]

Manganese oxide biomineralization is a social trait protecting against nitrite toxicity.

Christian Zerfaß[a,b], Joseph A. Christie-Oleza[a,b], and Orkun S. Soyer[a,b,*]

[a] School of Life Sciences, BBSRC/EPSRC Warwick Integrative Synthetic Biology Centre (WISB), University of Warwick, Coventry, CV4 7AL, UK.

[b] Warwick Integrative Synthetic Biology Centre (WISB), University of Warwick, Coventry, CV4 7AL, UK.

Supplementary Information

This supplementary text comprises the supplementary figures and table referred to in the main text.

Supplementary Table:

Protein Name	UniProt Accession Number
Catalase-peroxidase	A6FK08
Cytochrome c peroxidase	A6FV81
Glutathione peroxidase	A6FTQ6
Alkyl hydroperoxide reductase AhpD	A6FTV0
Animal haem peroxidase	A6FV45
Heme peroxidase	A6FKA4
Heme Peroxidase	A6FV44
Animal haem peroxidase	A6FKA5
Animal haem peroxidase	A6FKA6
Hemolysin-type calcium-binding region	A6FKB3
Hemolysin-type calcium-binding region	A6FV55
Hemolysin-type calcium-binding region	A6FN32

Table S1. List of AzwK-3b proteins with predicted peroxidase activity. The uniprot database (www.uniprot.org) was queried for "peroxidase" in *Roseobacter sp.* AzwK-3b, organism ID 351016, Proteome UP000004119. Note that this dataset is derived from an unassembled whole genome shotgun sequencing according to the uniprot homepage information, and hence the analysis presented here shall serve as an orientation. In the table, the protein names of interest (i.e. associated peroxidase activity predicted) and uniprot accession numbers of the entries are listed.

Supplementary Figures:

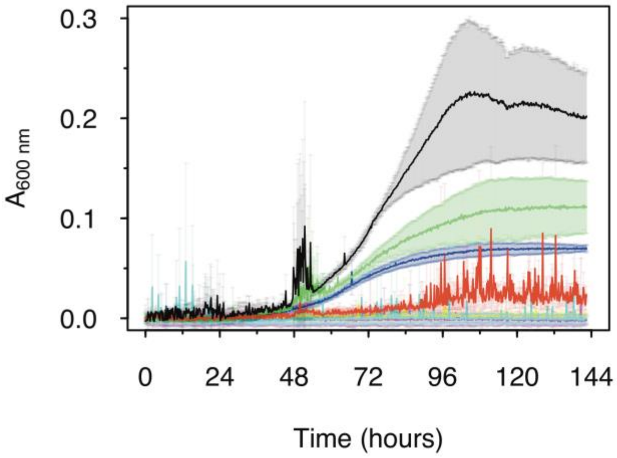

Figure S1. Vitamin-dependance of *Roseobacter sp.* AzwK-3b. Growth curves for AzwK-3b are shown for modified artificial sea water media (see Table 1) supplemented with different vitamin cocktails; a five vitamin cocktail with Pyridoxine, Riboflavin, Biotin, Thiamine, and Nicotinic acid (black curve), the same but without Pyridoxine (green), Riboflavin (blue), or Biotin (red). When Thiamine (light blue) or Nicotinic acid (yellow) were excluded, there was no observed growth. The non-vitamin negative control is shown in magenta.

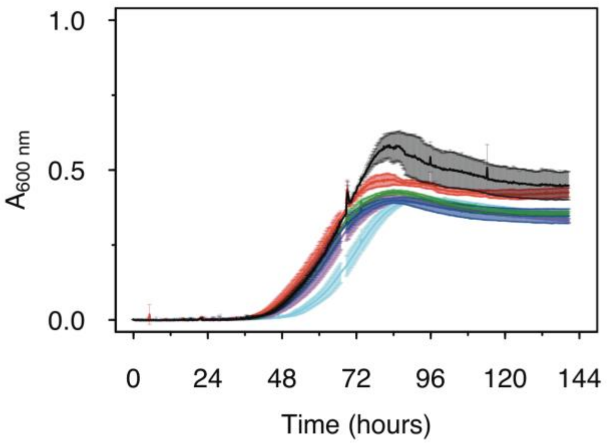

Figure S2. Growth of *Roseobacter sp.* AzwK-3b at different NaCl concentrations (all at 25 mM acetate). Growth curves from most conditions overlay considerably, and only the 150 mM condition (light blue) is notably slowing down AzwK-3b growth. The colours indicate the NaCl-conditions as follows: 428 mM (black; artificial seawater default), 350 mM (red), 300 mM (dark green), 250 mM (blue), 200 mM (purple, default in defined AzwK-3b medium used here), 150 mM (light blue).

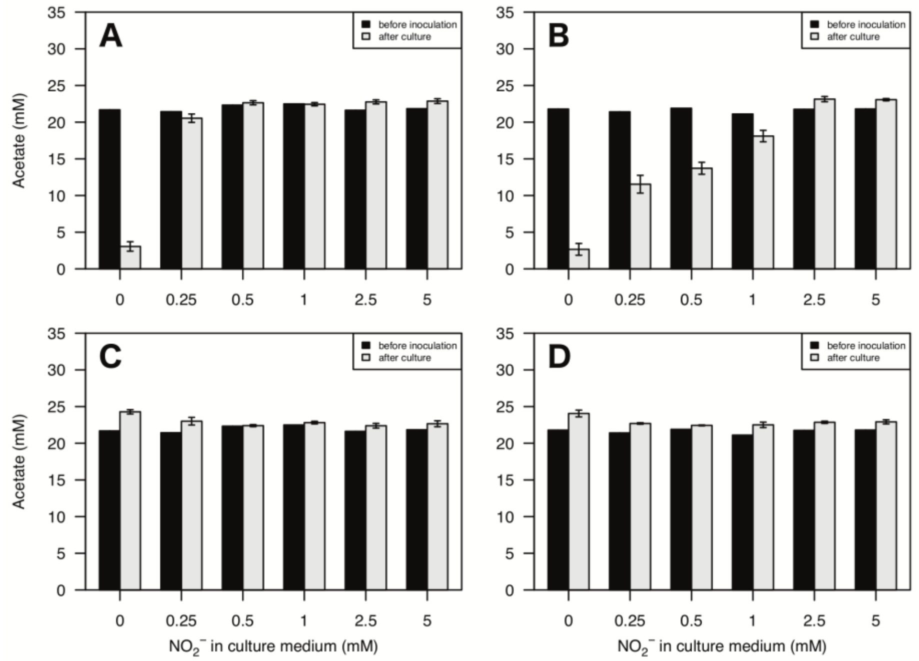

Figure S3. Acetate consumption in nitrite-inhibited cultures. The cultures presented in Figure 2 of the main text of the manuscript were analyzed by ion chromatography to determine the acetate concentration before (black bars) and after (white bars) the AzwK-3b growth experiment. The individual figures show the results for **A)** AzwK-3b inoculated, manganese-free cultures; **B)** AzwK-3b inoculated, Mn^{II}-supplemented cultures, **C)** non-inoculated, manganese-free cultures, **D)** non-inoculated, Mn^{II}-supplemented cultures. The concentration of nitrite added to the culture medium is indicated on the x-axis, with "0" indicating the nitrite-free (positive) controls.

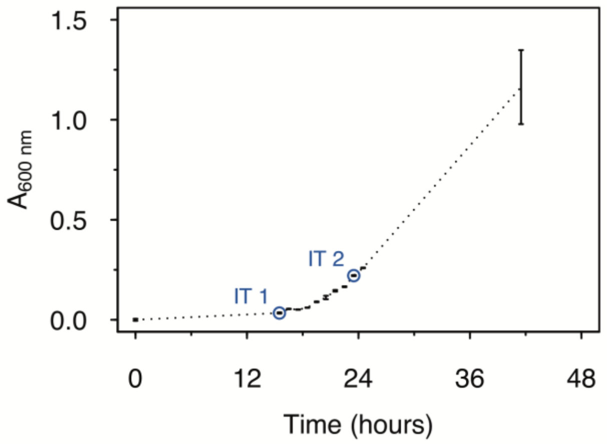

Figure S4. Pre-cultures used for the nitrite assay shown in Figure 3. Three 50 ml pre-cultures (no manganese, no nitrite) were grown and sampled at different time points as inoculum (mixing equal volumes of all three cultures). This inoculum was then 2x diluted with fresh medium, thereby introducing the +/- NO_2^- and +/- $Mn^{2+}Cl_2$ supplement (see Figure 3 in publication). Two time-points were chosen for preparation of an inoculum: IT 1 and IT 2, both of which were in the first third of the exponential phase.

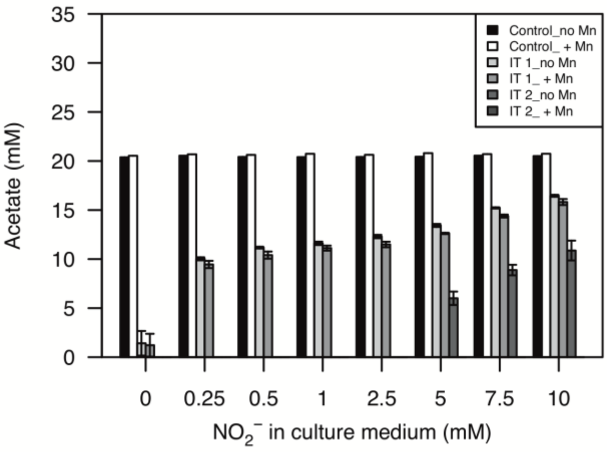

Figure S5. Acetate consumption confirms growth differences. The cultures presented in Figure 3 were subjected to ion chromatography before and after culture of AzwK-3b. The manganese free and Mn^{II}-supplemented (non-inoculated) medium acetate-levels are shown in black and white, respectively, and the earlier (IT 1) and later (IT 2) inocula +/- Mn^{II} are shown in increasingly dark grey bars as noted in the figure legend. Note that the Mn^{II}-supplemented IT 2 culture did not contain acetate above the detection threshold in any of the nitrite-conditions and hence no bars are shown. The concentration of nitrite added to the culture medium is shown on the x-axis, with “0” indicating the nitrite-free (positive) controls.

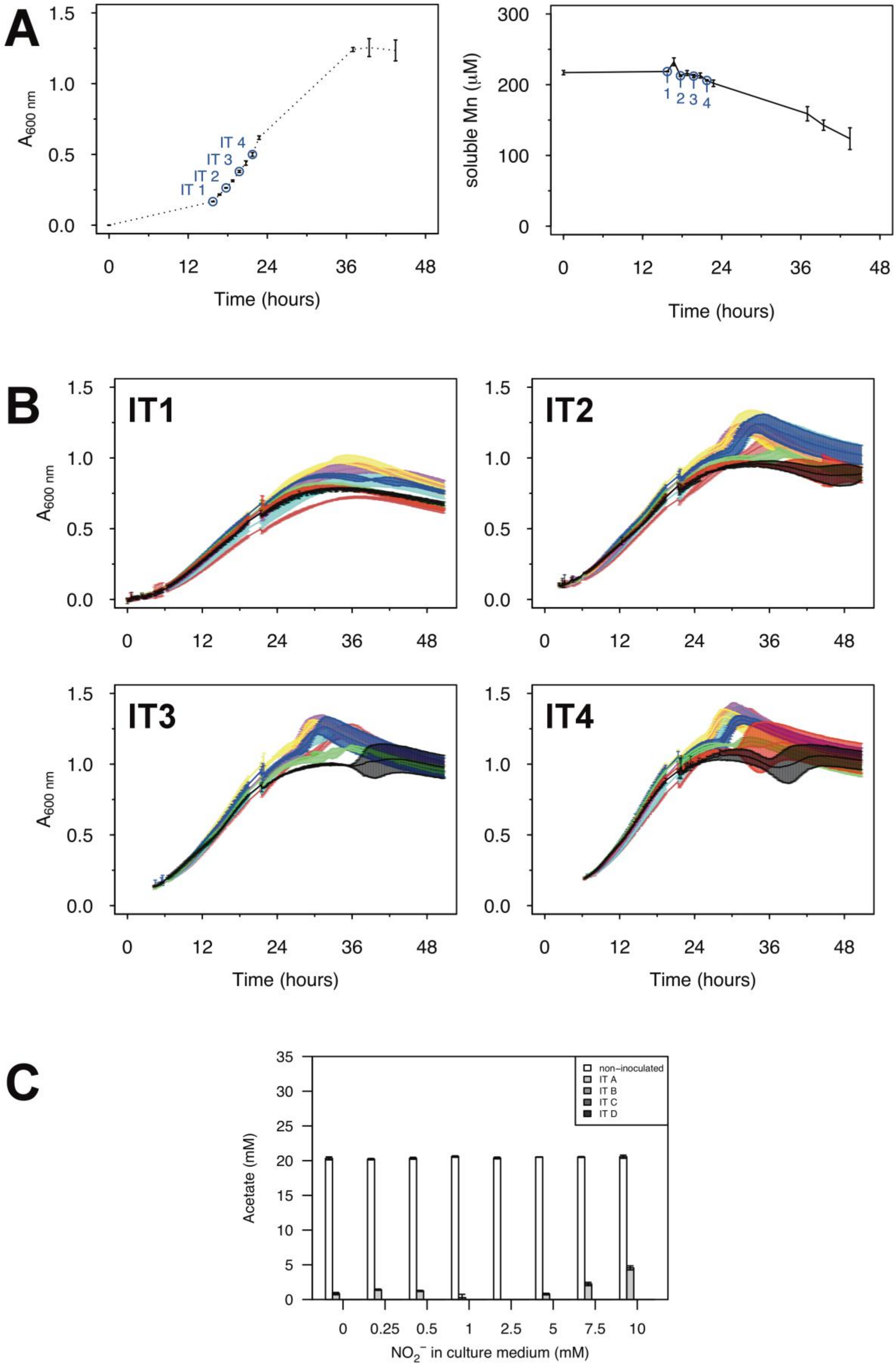
A
A600 nm
IT 1
IT 2
IT 3
IT 4
Time (hours)
soluble Mn (μM)
1 2 3 4
B
IT1
IT2
IT3
IT4
C
non-inoculated
IT A
IT B
IT C
IT D
Acetate (mM)
NO2− in culture medium (mM)

Figure S6. Supplementation of AzwK-3b pre-cultures with manganese helps overcoming the growth-inhibiting effects of nitrite. **(A)** A 50 ml pre-culture (200 µM $Mn^{II}Cl_2$, no nitrite) was grown (left) and manganese oxidation determined by quantifying soluble (residual) Mn^{II} (right) (see Methods in the main text for quantification and experiment details). This culture was sampled at different time points (IT 1-4) as inoculum. This inoculum was then 2x diluted with fresh medium, introducing $Mn^{II}Cl_2$ supplement and +/- NO_2^-. The growth curves of these inoculated cultures is shown in **(B)**. The nitrite concentrations were: Black – control no nitrite. Red – 0.25 mM nitrite. Green – 0.5 mM nitrite. Blue – 1 mM nitrite. Yellow – 2 mM nitrite. Magenta – 5 mM nitrite. Light blue – 7.5 mM nitrite. Dark red – 10 mM nitrite. **(C)** shows the acetate before (white bars) and after the growth of AzwK-3b at the different nitrite-concentrations (increasing greyscale for IT 1-4). The concentration of nitrite added to the culture medium is shown on the x-axis, with "0" indicating the nitrite-free (positive) controls. No bars are seen for IT 2-3, since acetate was below the detection limit in these samples.

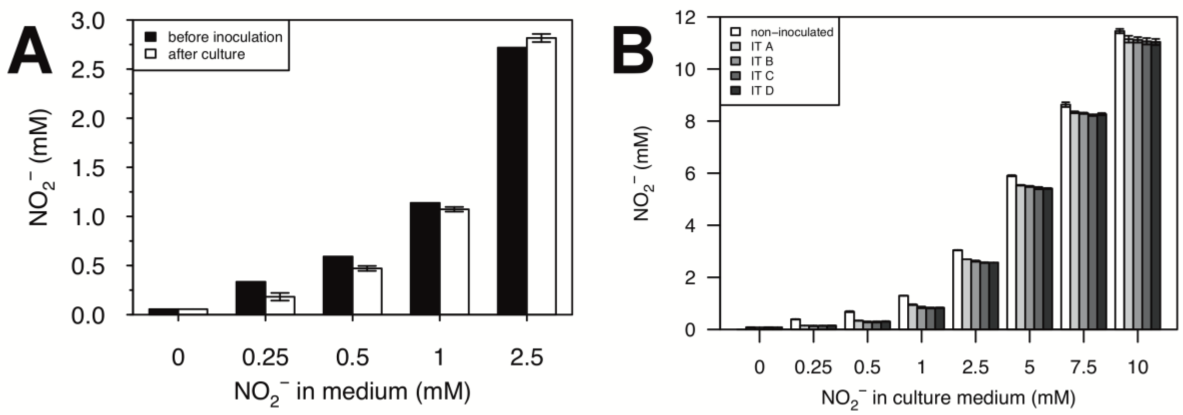

Figure S7. Nitrite conversion by AzwK-3b. Cultures reported in Figure 2 (non-dense inoculum) **(A)** and Figure S6 (inoculated from MnII-supplemented pre-culture) **(B)** were analyzed. In **(A)**, only the conditions up to 2.5 mM nitrite, in which no growth was seen anymore (see Figure 2), are reported. Nitrite was quantified before (black) and after (white) culturing AzwK-3b. In **(B)**, white bars indicate the nitrite before culture, and the cultures of different inoculation time point (IT 1-4) are indicated by bars in increasing greyscale. The concentration of nitrite added to the culture medium is shown on the as x-axis, with “0” indicating the nitrite-free (positive) controls.

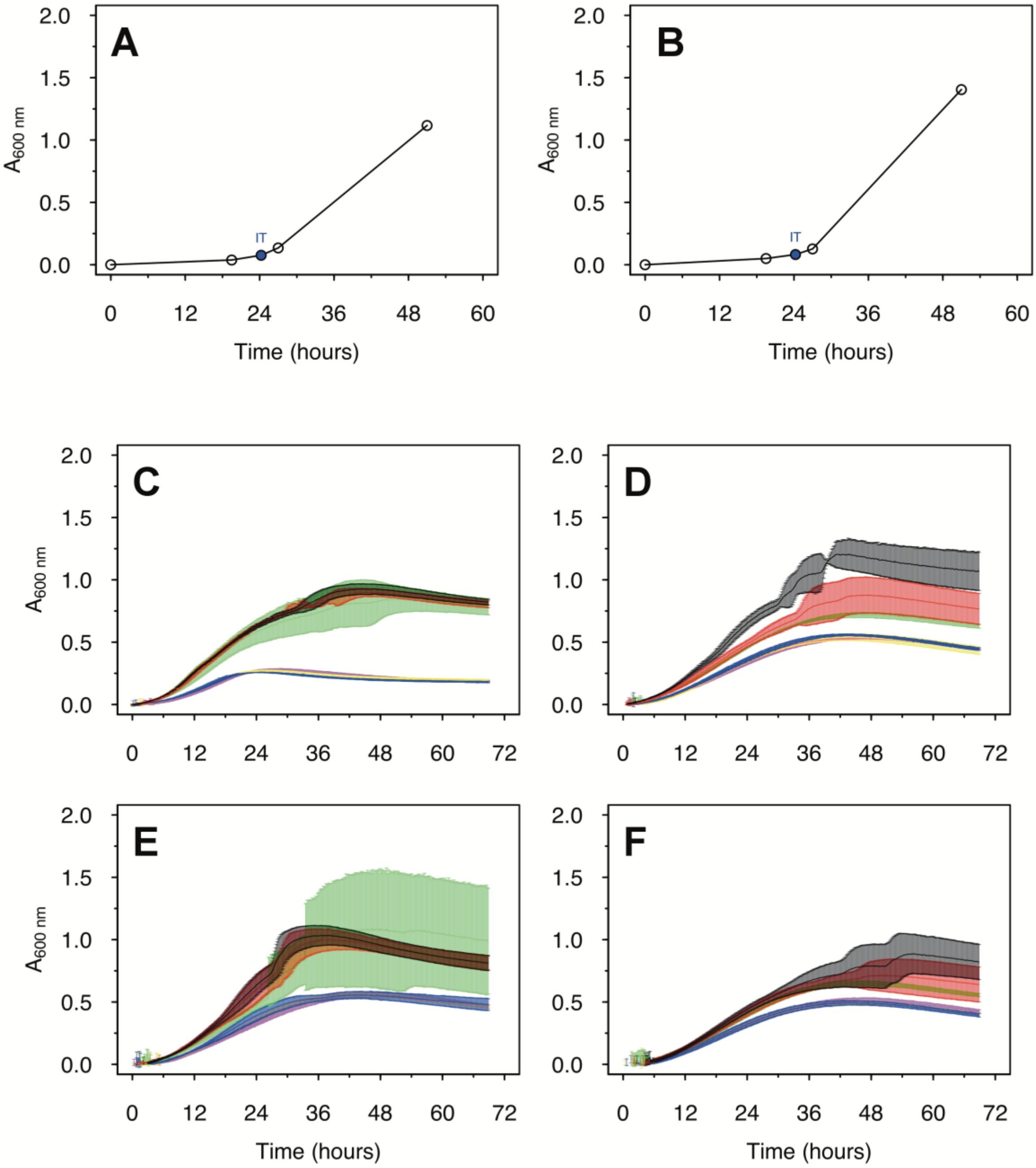

Figure S8. Effect of hydrogen peroxide on AzwK-3b. Growth curves from pre-cultures grown without **(A)** or with **(B)** 200 µM Mn^{II} supplement. Different inocula were drawn from these cultures at the time-points labeled "IT". These inocula were used for a hydrogen peroxide exposure assay as follows: Cultures **(C)** and **(D)** were prepared from a manganese-free pre-culture, and were subsequently grown without **(C)** or with **(D)** 200 µM Mn^{II} supplement. Cultures **(E)** and **(F)** were prepared from the Mn^{II}-supplemented pre-cultures, and **(F)** received additional manganese supplement. Note that in this case, the Mn^{II} supplement was in total 300 µM (due to the manganese in the pre-culture), and in **(E)** the concentration of manganese

was 100 µM. The nitrite-free controls containing 0, 50, and 100 µM H_2O_2 are shown in black, red, and green, respectively. Cultures with additional 5 mM nitrite, and containing 0, 50, and 100 µM H_2O_2 are shown in blue, yellow and purple, respectively.
